# Pixel-CO_2_ laser for the treatment of stress urinary incontinence

**DOI:** 10.1007/s10103-021-03353-7

**Published:** 2021-08-12

**Authors:** Agnieszka Aleksandra Nalewczynska, Michael Barwijuk, Piotr Kolczewski, Ewa Dmoch-Gajzlerska

**Affiliations:** 1 Department of Obstetrics and Gynecology Didactics, Faculty of Health Sciences, Medical University of Warsaw, 14/16 Litewska Street, 00-575 Warsaw, Poland; 2 Estebelle Clinic Sarmacka, 13/129, 02-972 Warsaw, Poland; 3 Warsaw, Poland; 4 Medifem Clinic, 488th Pulawska Street, 02-884 Warsaw, Poland; 5 Center for Reproductive Health, Institute of Mother and Child, 17a Kasprzak Street, 01-211 Warsaw, Poland; 6 Esthegyn Clinic, 7th Mazurska Street, 71-899 Szczecin, Poland; 7 Department of Forensic Medicine, Pomeranian Medical University, 72nd al. Powstańców Wielkopolskich Street, 70-111 Szczecin, Poland

**Keywords:** Stress urinary incontinence, Vaginal CO2 laser, Fractional-pixel laser, Quality of life, Fractional laser, Pixel CO2 laser

## Abstract

**Abstract:**

The aim of this study was to assess the safety and efficacy of a minimally invasive pixel-CO_2_ laser procedure for the treatment of stress urinary incontinence (SUI). This was a prospective, open-label study with a cohort of 59 women. Patients were treated intravaginally with a fractional/pixel CO_2_ laser every 4–6 weeks for a total of three treatments and assessed at 3, 6, and 12 months. Evaluation tools included a Sandvik severity score based on a validated questionnaire, 1-h pad test, vaginal health index score (VHIS), validated female sexual function index (FSFI), patient’s impression of disease severity (PGI-S), global impression of improvement (PGI-I), and the short-term pelvic floor impact questionnaire (PFIQ-7) to assess improvements in quality of life. Reduction in SUI severity was noticed throughout the duration of the study, as compared to the baseline in which 2% of the patients were defined as “slight,” 73% “moderate,” and 25% “severe.” Gradual improvement of symptoms resulted in redistribution of severity score and the best outcome observed between 3 and 6 months. Sanitary pad weight declined from an average of 35.45 g per day at baseline to 12.47 g at the 3rd treatment, and increased to 23.06 g at 12 months. Vaginal acidity changes showed a similar pattern. No serious adverse events were reported. Pixel-CO_2_ laser is safe and effective for treating SUI. Additional maintenance treatments should be considered during the 6–12-month post-treatment period in order to maintain the beneficial effects.

**Brief summary:**

Pixel-CO_2_ laser is a safe and effective treatment for SUI. Maintenance treatments should be considered at 6–12 months.

## Introduction

The first clinical use of the ablative CO_2_ laser was to treat erosions on the uterine cervix [1], and technological improvements diversified treatment options from an ablative tissue elimination with focused laser beams, to microablative tissue stimulation with fractional beams [2–5]. The role of CO2 laser as a standard treatment for genitourinary syndrome of menopause (GSM) or stress urinary incontinence (SUI) is still controversial although it is cleared by governing bodies such as the Food and Drug Administration (FDA) for inducing tissue effects such as ablation, vaporization, excision, incision, and coagulation of soft tissue. Professional bodies such as the North American Menopause Society (NAMS) and the International Society for the study of Women’s Sexual Health (ISSWSH) in a consensus recommendation have stated that when treating GSM in women that are high risk for breast cancer, the microablative fractional CO2 laser, or other non-ablative energy-based treatments, can offer a potential advantage over pharmacologic therapies [6]. Other professional organizations such as the European Society for Sexual Medicine (ESSM) and the International Urogynecological Association (IUGA) issued committee opinions stating that therapeutic advantages of nonsurgical laser-based treatments can only be recommended after robust clinical trials that will demonstrate their short-/long-term benefits and complication profile [7, 8].

As the future of mid-urethral slings remains unclear, the need for alternative minimally invasive treatments is justified. A growing body of evidence is now available on the use of non-ablative Er:YAG laser for the improvement of SUI symptoms [9]. Moreover, data is accumulating on the comparison between this new modality and conventional treatments such as sling procedures [10] and topical estrogen [11, 12]. Other studies outline predictive factors of success, and duration of improvement [13, 14], though, sham control studies are still required.

CO_2_ lasers can be designed to produce a fractional pattern in two very different ways: The first involves a galvanometric scanning motor that allows micro-beams to be lased one after the other, creating a fractional pattern for rapid coverage while the second technology involves splitting the laser beam using an optical element to micropixels that target the skin simultaneously. When lasing using a motorized scanner, each microbeam is of high power and short pulse width (HP-SP) expressed in a highly ablative effect on the skin tissue [15]. By splitting the laser beam optically to several micropixels, the micropixels are of low power and long pulse width (LP-LP) compared to the scanner and therefore create a more coagulation effect surrounding the ablated area.

Recent publications with various treatment protocols highlighted the effective use of fractional-scanning CO_2_ laser [16–19], and a fractional-pixel CO_2_ laser [20] for SUI symptoms; however, randomized trials are urgently needed.

This prospective observational study is aimed at providing data on the long-term outcome of a pixel CO_2_ laser for the treatment of mild, moderate, and severe SUI symptoms.

## Materials and methods

### Study design and characteristics of the study participants

This was a prospective, open-label cohort study conducted in a secondary care facility. Female patients were referred from primary care physicians, and if they met the inclusion/exclusion criteria, they were recruited consecutively between December 2018 and February 2019. The study was approved by the institutional review board, human ethics committee of the faculty of Health Sciences, the University of Warsaw, Poland (KB/210/2018, dated Nov. 2018). A total of 60 women were enrolled and treated at the Estebelle Clinic, Warsaw. Fifty-nine of them completed three treatment sessions with 4–6 weeks intervals, and 3-, 6-, and 12-month follow-up visits. Only one patient did not attend the 6-month follow-up, a lower-than-expected dropout rate, strengthening the final results from this study.

All study subjects approved the protocol and guidelines which were available in the local language, by signing a written consent. Age range of the treated patients was 30–75 years old and the severity of SUI at the entry screening, which was the predominant symptom in all patients, was defined as mild, moderate, or severe, according to the Sandvik score [21]. Comorbidities included hypertension (n = 11), diabetes (n = 3, all hypertensive), and 5 patients defined as smokers.

Exclusion criteria comprised active vaginal infection, urge or overflow incontinence, pelvic organ prolapse (POP) ≥ grade III, BMI ≥ 40, previous surgical intervention for SUI, patients on antidepressants, α-adrenergic or anticholinergic medications, history of immune system diseases, and any other reasons that may compromise safety. Normal Pap smear within the last two years was confirmed.

At baseline and each follow-up session, we employed the 1-h pad test and validated questionnaires were completed, including Sandvik score to evaluate the severity of SUI, and vaginal health index score (VHIS) which was recorded by measuring vaginal elasticity, fluid volume, pH, epithelial integrity, and moisture using a scale of 1–5. Sexual function at each time point was subject-reported, using the validated female sexual function index (FSFI), and patient’s impression of disease severity (PGI-S) questionnaire on a 4-point scale, and subjective assessment was based on PGI-I, which demonstrated global impression of improvement. Pelvic floor impact questionnaire-short form (PFIQ-7) was used to assess how changes of bladder or vaginal symptoms affected the patients’ quality of life.

## Study intervention

The fractional microablative pixel CO_2_ laser system (FemiLift™, Alma Lasers, Israel) emits light at a wavelength of 10.6 μm via a dedicated vaginal probe. The laser intensity was adjusted according to patient’s tolerability and the level of vulvo-vaginal atrophy (VVA), ranging from 70 to 120 mJ/px. The laser beam is fractionated into 81 “px” per cm^2^ at each emission, and the expected depth of microablation varies between 300 and 500 μm with increased thermal margins of 150–200 μm (20,21). Following introitus lubrication with “baby oil”, and gentle insertion of the laser probe, the lasing procedure is performed by rotating the probe clockwise to cover the entire vaginal wall (360°) and then this maneuver is repeated three times (“passes”). Patients were instructed to avoid intense physical exercise as well as vaginal douches or lubricants, and to abstain from sexual intercourse for 5 days post-treatment.

## Statistics

Statistical analysis was performed using MedCalc statistical software (version 19.4.1) and graphs were produced by Excel. All paired t-test tests were 2-tailed and a P-value < 0.05 or less was considered statistically significant.

## Results

Fifty-nine volunteering women with clinically confirmed SUI were included in the study and received 3-px CO_2_ treatments, 1 month apart. Assessments were performed before each treatment session and at 6- and 12-month follow-ups.

Demographics of patients are summarized in Table 1. The patients’ mean age was 51.0 ± 1.4 (range: 32–70), parity was 1.7 ± 0.1 (0–3), mean BMI was 26.1 ± 0.4 (19–34). The group consisted of 68% high school education graduate women and 32% were academics. The following figures highlight the pattern of improved urinary symptoms following the described treatment protocol.

**Table 1:** Demographic characteristics and medical information of the involved patients

Number of patients	59	
Age (years) (mean ± SEM, range)	51.0 ± 1.4	32–70
BMI (kg/m^2^) (mean ± SEM, range)	26.1 ± 0.4	19–34
Number of deliveries (mean ± SEM, range)	1.7 ± 0.1	0–3
Number of vaginal deliveries (mean ± SEM, range)	1.1 ± 0.1	0–3
Number of cesarean deliveries (mean ± SEM, range)	0.6 ± 0.1	0–3

**Fig. 1:**
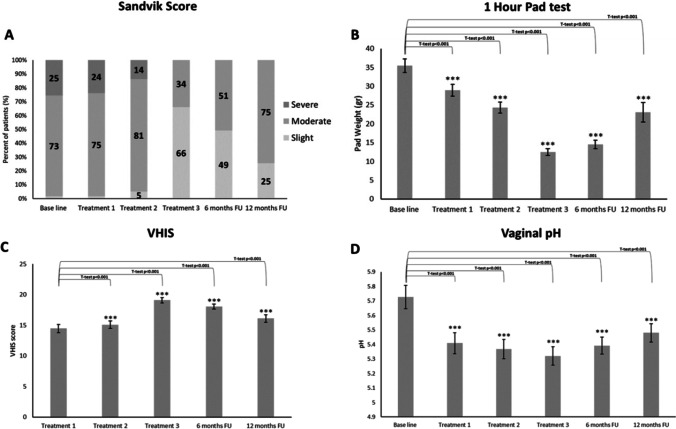
Sandvik’s SUI severity score (**A**), vaginal health score (**C**), pad test and pH (**B** and **D**): changes during the entire 12 months, treatments, and follow-up

Severity of SUI symptoms monitored according to the Sandvik index score revealed significant changes as compared to baseline where 2% were defined as slight, 73% moderate, and 25% severe (Fig. 1A). Gradual improvement of symptoms resulted in redistribution of the severity groups. Many patients in the moderate score group went down to the slight score group and the severe to the moderate score group towards the 6- and 12-month follow-up. One-hour pad test (Fig. 1B) documented a similar pattern in which the best outcome as compared to baseline was at the 3rd treatment session with gradual decline at 6–12-month follow-up (12.4 ± 0.8 g to 14.47 ± 1, 23 ± 2.6 g, respectively, *p* < 0.001).

**Fig. 2:**
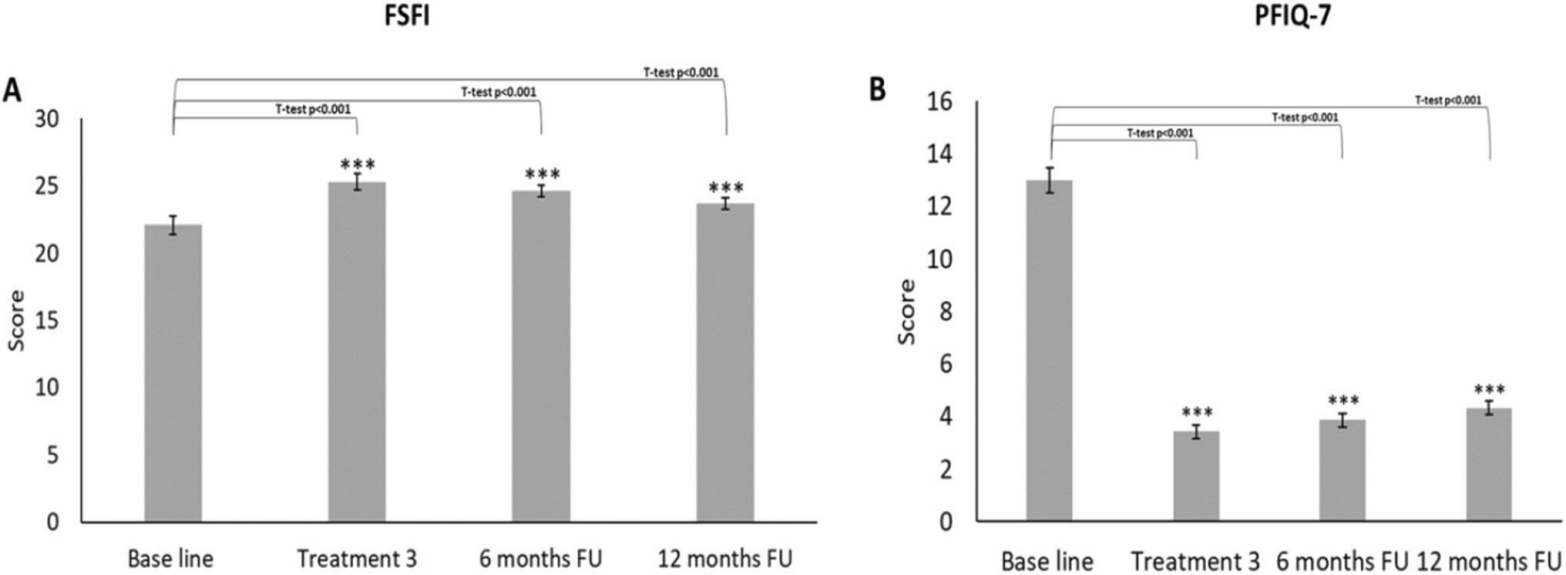
FSFI and PFI-Q 7 changes during the entire 12 months, treatments, and follow-up

Vaginal elasticity, fluid volume, vaginal pH, epithelial integrity, and moisture are integrated in the VHI score, and changes are demonstrated in Fig. 1C, D. The VHI score showed significant improvement of the index in the 3rd treatment and in the 6-month follow-up (19.0 ± 0.4 and 18.0 ± 0.4 respectively, *p* < 0.001). The vaginal pH score, as an objective parameter, showed significant and consistent improvement (increase) in the acidity with a similar pattern of changes when compared to the other parameters included in VHIS (5.4 ± 0.1 to 5.4 ± 0.1 respectively, p < 0.001). Validated female sexual function index (FSFI), and the pelvic floor impact questionnaire (PFIQ-7) used to assess how changes of bladder or vaginal symptoms affect their activities, detailed a similar pattern of self-reported parameters (Fig. 2A, B).

**Fig. 3:**
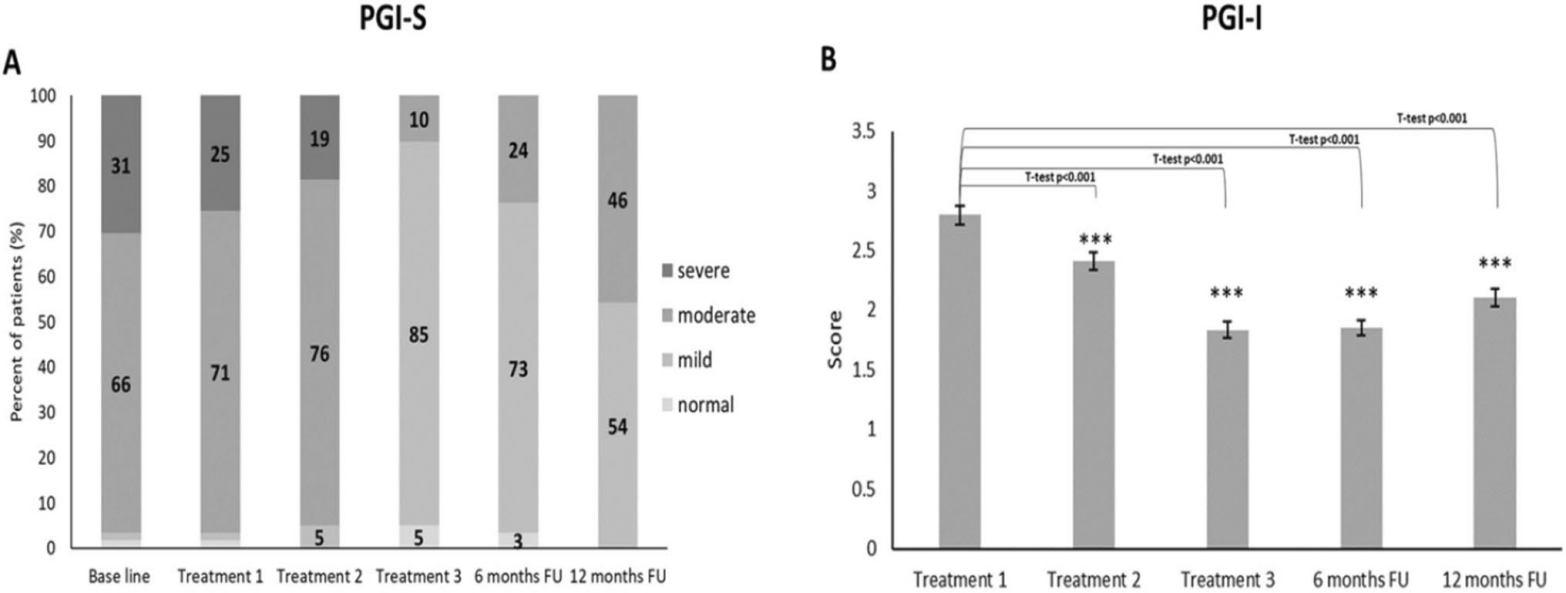
PGI-S and PGI-I changes during the entire 12 months, treatments, and follow-up

Patient’s impression (PGI-S) questionnaire on a 4-point scale validated the severity of SUI and another subjective assessment, PGI-I, described how the urinary symptoms changed along the follow-up time axis. As displayed in Fig. 3A, B, the PGI-I showed the same score of 1.8 ± 0.1 at the 3rd treatment and 6-month follow-up and remained statistically significant at 12-month follow-up (2.1 ± 0.1, *p* < 0.001), supporting the PGI-S results.

Furthermore, objective parameters such as pad test and pH were also analyzed according to BMI and patient’s age (data not shown). BMI was divided into three sub-groups. < 25 (n = 19), 25–30 (n = 34), and > 30 (n = 6). Improvement according to the pad test was found statistically significant in all groups (*p* < 0.01, *p* < 0.001, and *p* < 0.001, respectively). The pattern seen with the changes in pH was similar to that seen in the pad test (Fig. 1B). This shows an improvement until treatment 3 and then a slight reversion in the following months. Furthermore, the pH changes revealed a more significant outcome in the older age groups.

In addition, patient’s satisfaction from the treatment outcome, as well as toleration during treatment, was recorded on a 1–10 scale (with 10 being the most positive) with the 7-score mark dominating the feedback for both questions. Pain was also graded using a similar 1–10 scale where 1 was the least painful and 10 the highest level of pain. Fifty-one percent of patients rated their pain as level 3, 20% level 4, 14% level 5, and 10% pain level 2.

No serious adverse events were recorded during the procedures or the study period. Minor side effects related to the treatment included vaginal discharge (34%), swelling (15%), itching (11%), numbness (3%), and purpura (3%) which did not last more than 5 days. No hospitalization was needed.

## Discussion

This study describes the improvement of SUI symptoms following treatment with a fractional/pixel CO_2_ laser. Improved treatment outcome during the 12-month follow-up is clearly observed by inter-group changes as compared to pre-treatment severity, defined by the widely used Sandvik score (Fig. 1A). Gradual improvement reached the best outcome at the final treatment session and no one scored as severe. Redistribution almost equaled between the moderate and slight symptoms at 6 months, with a slow decline towards the baseline after 12 months. The slight and moderate groups still dominated the severity symptoms with no severe SUI as compared to the baseline. Two different objective parameters, 1-h pad test and vaginal pH (Fig. 1B and D) demonstrated an identical pattern. Subjective monitoring tools such as the female sexual function and pelvic floor impact questionnaire show similar patterns of improvement which support the objective assessment tools. Other subjective tools such as global impression of improvement and patient’s impression of disease on a 4-point scale (Fig. 3A, B) offer additional backup to the validity of this improvement pattern.

The results from objective parameters such as pad test and pH test were further analyzed according to patient’s age and BMI (data not shown). Statistically significant improvement in pad test is demonstrated in the normal BMI group and even a better outcome in the overweight group; however, there were only 6 women in the obese group which may distort the validity of the pad test outcome of this small subgroup. Vaginal acidity is related to hormonal status and normal pH is usually less than 4.5 during the reproductive age. Physiologic changes towards a less acidic environment (pH 5–6) is common amongst post-menopausal women. As such, the lack of significant changes in pH levels in the younger age group (under 50 years old) was expected. Acidity changes in this study were highly significant in the older age groups and the pattern of changes along the duration of the study identical to the other monitoring parameters described above (data not shown).

Other studies using a non-ablative Er:YAG laser [11] and microablative CO_2_ laser [22] described similar pH changes. The correlation between body mass index and the expected outcome of energy-based treatments is still controversial. Fistonic and Fistonic [23] concluded that treatment outcomes with Er:YAG laser is likely to be better in women with BMI < 23.3 kg/m^2^, which was below the average of their studied patients. Blaganje et al. used the same laser wavelength with a different fractional technology and concluded that BMI had no significant effect on the outcome [7], while Alcalay et al. who used pixel-CO_2_ laser excluded patients if their BMI was greater than 38 [20]. Interestingly, our results may indicate that the moderately overweight patients benefit from this treatment. However, the small number of subjects in these studies does not allow us to draw firm predictive conclusions about the relevance of BMI values for energy-based treatment options. These observations are highlighted for the scientific community to take into consideration once larger sample sizes studies with control groups will be designed. Similarly, the importance of age group distribution, which in our study starts at 30–40 years, should be investigated as results may differ in other studies in which the average age is higher.

Based on the majority of subjective and objective monitoring tools used in this study, the optimal outcome of improved SUI symptoms following fractional CO_2_ laser treatment was observed between the 3rd treatment session and the 6-month follow-up, and lasted for the duration of study. The pattern of a slow return towards pre-treatment symptoms is visible by all monitoring tools and may suggest the need for a touch-up session at around 9–12 months. Similar trends of improved SUI symptoms and slow return towards baseline levels between 6 and 12 months are reported by Alcalay et al. [20] following a similar treatment protocol of fractional/pixel-CO_2_ laser treatment with additional serial urodynamic monitoring. Dabaja et al. [17] described similar improvements following fractional/scanning-CO_2_ laser treatments around the 6-month follow-up time point.

There is an increasing amount of evidence supporting energy-based treatment protocols and tools for SUI. Isaza et al. [16] treated SUI with fractional/scanning CO_2_ laser and after four monthly sessions recommended annual “retouches” for three consecutive years. Multiple biopsies demonstrated epithelial thickening and better organized underlying connective tissue. Behina-Willison et al. [18] treated various severity groups of urinary disfunction with another fractional CO_2_ laser protocol and described the changes of the prevalence of SUI symptoms, both in pre- and post-menopausal women. The study showed that following three treatments, SUI symptoms improved in 80% of participants at 3 months (*p* < 0.01) and that these benefits persisted in 75% of participants at 12 months (*p* < 0.01).

Dabaja et al. [17] demonstrated improvement in SUI symptoms at 3 months, and a return to baseline towards the 6th month post-treatment follow-up. Another dedicated vaginal probe in which the microablative scanning CO_2_ laser was used for to treat SUI is described by Palacios et al. [19] where they treated a range of severity in urinary incontinence. The protocol included three, 4–6 weeks apart, treatment sessions of the entire vaginal wall with two additional passes of the anterior, sub-urethral wall and resulted in improved SUI and MUI symptoms. However, the short 4–6-week follow-up is not long enough to draw firm conclusions.

Ogrinc et al. [6] treated 175 women with SUI and mixed degrees of other urinary disorders with non-ablative Er:YAG laser technology and concluded that the treatment was the most effective for SUI patients. An improved outcome was assessed by an International Consultation of Incontinence questionnaire and an Incontinence Severity Index that both indicated a significant improvement for 6–12 months. Erel et al. [13] defined the preferred group of patients with SUI that may benefit from a non-ablative laser, to be the younger, pre- or post-menopausal women with normal BMI. Lin KL et al. [8] when treating women with mild-to-moderate SUI suggested that the mechanism of action responsible for the improvement of symptoms is related to a decrease in bladder-neck mobility.

A wide range of subjective improvements following various surgical interventions was reported by Imamura et al. [24], who graded the most effective interventions with an average probability of 97%, 76.1%, 67.7%, and 63.8%, for retropubic MUS, trans-obturator MUS, traditional sling, and open colposuspension, respectively. Subjective methods used in our study to assess improvement following the less invasive CO_2_ laser treatment resulted in a similar percentage range following 6 and 12 months.

The encouraging outcome of this study as detailed by the patient’s satisfaction and treatment tolerability indicates the potential for this technology as an ambulatory alternative to mesh implants which has come under scrutiny [25, 26]. The small number of minor adverse events in this study and reported with other energy-based treatment protocols for SUI favors this alternative approach. The overall concept of energy-based treatments for age-related vaginal symptoms is still questionable although the outcome of this study strengthens the validity of other recent publications comparing vaginal laser therapy to vaginal estrogen therapy for GSM [11, 12]. Randomized double-blinded sham-controlled trials for GSM such as the recent one by Purim et al. [27] are urgently needed. Studies using non-invasive optical monitoring of histological changes in the epithelium and lamina propria of the vaginal wall may provide additional support to the efficacy of this treatment modality [28].

The results presented in this manuscript support the recently published study in which a similar pixel CO_2_ laser technology is used for the same indications with an identical treatment protocol, although both studies lack a control arm [20]. In July 2018, the FDA delivered a warning letter to industries, focusing on safety and efficacy issues of energy-based devices for vaginal applications [29]. The scientific evidence behind treating peri-vaginal indications is continuing to grow, but large sample size sham-controlled studies are still needed.

## Conclusions

Pixel-CO_2_ laser is safe and effective for treating SUI. Additional maintenance treatments should be considered during the 6–12-month follow-up period in order to maintain the beneficial effects.
